# Case Report: Double chimney in valve-in-valve procedures for high-risk coronary obstruction

**DOI:** 10.3389/fcvm.2023.1270782

**Published:** 2023-12-04

**Authors:** Selma T. Cook, Mario Togni, Stéphane Cook

**Affiliations:** Cardiology, University and Hospital, Fribourg, Switzerland

**Keywords:** transcatheter aortic valve implantation (TAVI), coronary artery obstruction (CAO), chimney technique, valve-in valve, VIVID classification

## Abstract

The chimney technique has been utilized to minimize the risk of coronary artery obstruction during valve-in-valve procedures. Here, we present a case involving an 89-year-old female patient with low coronary ostia, severe aortic regurgitation, and intractable heart decompensation caused by degenerated aortic bioprosthesis. The patient underwent a successful transcatheter aortic valve implantation procedure using the chimney technique in both coronary ostia.

## Introduction

Bioprosthetic heart valves possess limited durability (1). The transcatheter aortic valve implantation (TAVI)-in-valve represents an alternative to surgical redo procedures in instances of surgical aortic bioprosthesis degeneration. Notably, it facilitates the restoration of nearly normalized opening area, albeit with a low operative risk. However, TAVI-in-valve entails a specific hazard of coronary occlusion. This risk arises from the presence of degenerated valve leaflets, which can impede the TAVI valve scaffold and exclude the coronary arteries. It is particularly pronounced in cases where the distance to the annulus is short (<10 mm) and the aortic sinuses are narrow. The risk also depends on the type of bioprosthesis and the choice of TAVI device.

In scenarios involving a considerable risk of coronary obstruction, two techniques may be considered. The first technique is known as “Bioprosthetic Aortic Scallop Intentional Laceration to prevent Iatrogenic Coronary Artery obstruction,” or BASILICA, which entails creating a laceration of the valve leaflets near the coronary ostium before the placement of the TAVI. The second technique is referred to as the “chimney” technique, where a coronary extension is fashioned parallel to the TAVI stent through the use of a coronary stent.

## Case report

An 89-year-old woman, with a history of arterial hypertension, moderate to severe renal insufficiency [chronic kidney disease stage 3b, estimated glomerular filtration rate (eGFR) 37 ml/min], and underwent aortic valve replacement (AVR) without bypass surgery seven years ago, presented with non-ST-elevation myocardial infarction (NSTEMI) accompanied by atrial fibrillation and heart failure. The NT-pro-BNP level was measured at 21,090 ng/L.

Coronary angiography revealed a 70%–90% stenosis in the left anterior descending artery (LAD), occlusion of the first diagonal branch, and a 50%–70% stenosis in the mid circumflex artery. These three lesions were treated with percutaneous coronary intervention (PCI) using two drug-eluting stents. The left ventricular ejection fraction (LVEF) was measured at 45% with lateral hypokinesia. A transthoracic echocardiogram demonstrated severe regurgitation of the 19 mm Trifecta bioprosthesis, with endocarditis ruled out.

**Figure 1:**
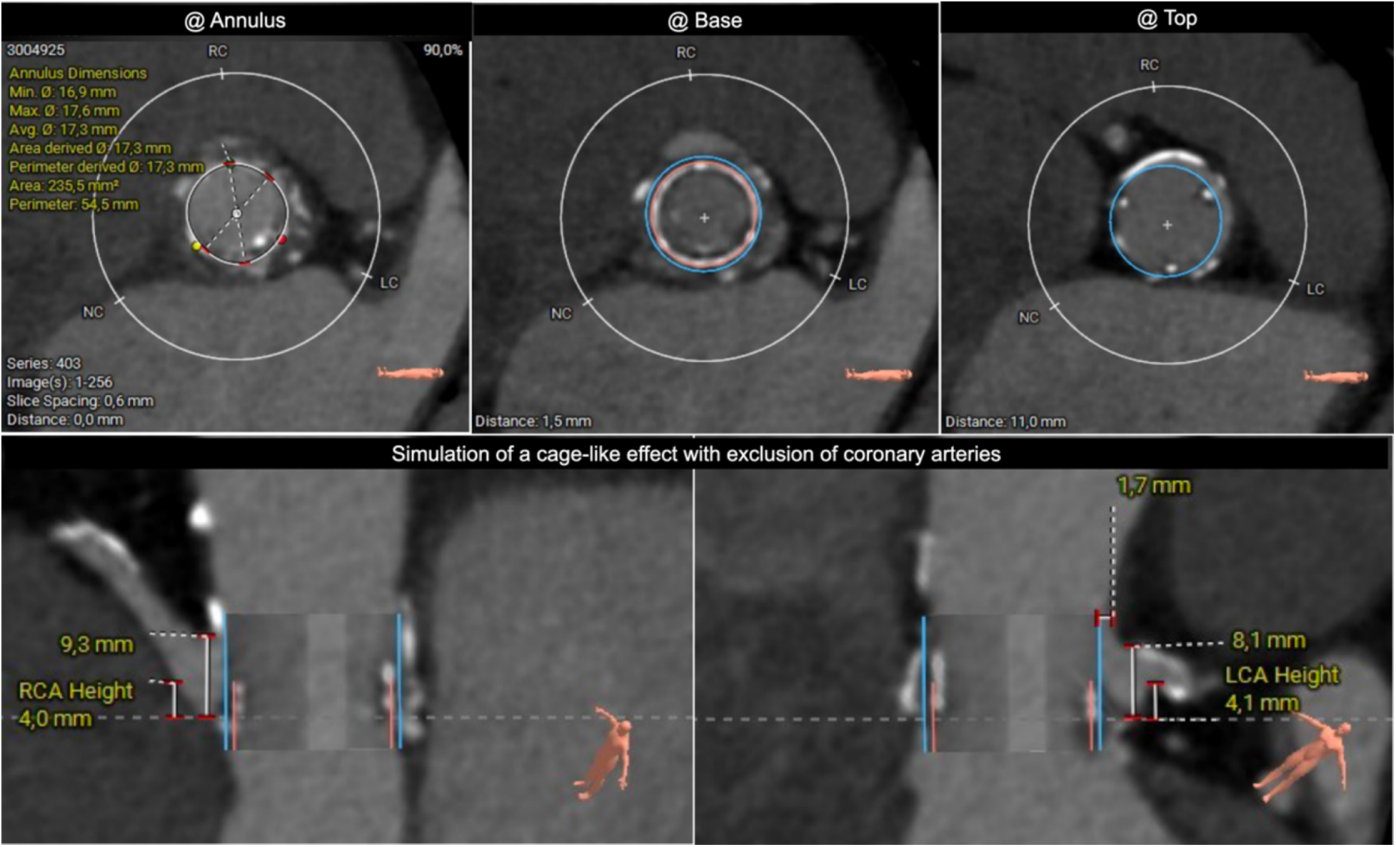
Pre-TAVI simulation of cage-like effect with exclusion of coronary arteries.

During the early out-of-hospital course, the patient experienced two readmissions due to cardiac decompensation. An angio-CT was performed to evaluate the feasibility of a TAVI procedure. However, due to the low position of the ostia, there was a high risk of coronary artery obstruction (CAO) during valve placement, making TAVI potentially risky (Figure 1). Valve-to-coronary (VTC) and valve-to-sinotubular junction (VTSTJ) distances were measured following virtual implantation of the TAVI planned. The measurements (VTC-LCA: 5.4 mm, VTC-RCA: 1.2 mm, VTSTJ-LCA: 0 mm, VTSTJ-RCA: 0 mm) indicated that the anatomy was at a very high risk of sequestration [Valve-in-Valve International Data (VIVID) class III c on both sides]. Considering the patient’s recurrent episodes of cardiac decompensation, the possibility of performing a TAVI procedure with BASILICA was deliberated. However, our experience with this technique is limited, and we deemed the risk of hemodynamic deterioration associated with the procedure, involving two of the three leaflets, to be excessive in this patient already on the hemodynamic margin. Consequently, we opted for an alternative approach, employing a double chimney technique to maintain hemodynamic stability throughout the procedure. Furthermore, to ensure optimal conditions for the procedure, it was pre-emptively decided to administer general anesthesia. This facilitated maximum vasodilation (beneficial in aortic regurgitation) and positive ventilatory pressure (due to pulmonary edema). This decision was made before the procedure and was comprehensively explained to the patient and her children.

**Figure 2:**
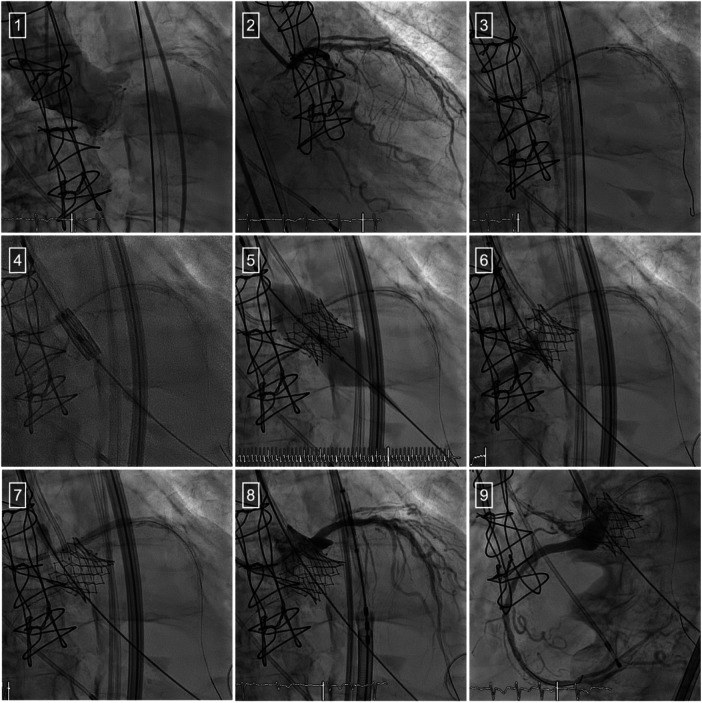
Transcatheter aortic valve implantation using the chimney technique. (1) Aortography to visualize the location of the ostia of the right and left coronary arteries. (2) Coronary angiography of the left coronary artery. (3) Coronary protection using the chimney technique. (4) Placement of the Edwards Sapien III valve. (5) Valve deployment. (6) Placement of the stent in the RCA. (7) Placement of the stent in the LCA. (8) Coronary angiography of the LCA. (9) Coronary angiography of the RCA.

The aortic valve implantation was carried out under general anesthesia, along with the placement of a pacemaker through the right jugular vein (Figure 2). Following the insertion of two Sherpa Active 6F femoral catheters [Q3.5 in the left coronary artery (LCA) and JR4 in the right coronary artery (RCA)], coronary protection was ensured by pre-emptively positioning a Sion Blue 0.014" guidewire in the LAD and RCA. For the deployment of an Edwards Sapien 3 Ultra 20 mm valve, a Safari ES guidewire was utilized to align with the Trifecta bioprosthesis in the aortic position. Concurrently, coronary stents were positioned to align with the upper section of the valve frame. The valve was implanted with overdrive pacing at 180 bpm, immediately followed by the placement of a 3.5/28 mm-Xience Skypoint (Abbott vasc.) stent for the LCA and a 3.5/30 mm-Onyx Resolute (Medtronic) stent for the RCA. Both proximal ends were post-dilated at 20 bars. Aortography confirmed a stable position of the bioprosthesis with no aortic regurgitation and patent coronary arteries. The patient was discharged from the hospital after a 4-day stay.

At the 3-month follow-up, the patient was free of symptoms. The electrocardiogram showed normocardic atrial fibrillation. Transthoracic echocardiography (TTE) revealed excellent function of the prosthetic valve with a mean gradient of 18 mmHg and preserved opening. The LVEF remained stable at 45%.

## Discussion

While TAVI procedures are typically not considered for severe native aortic valve insufficiency, they play a significant role in cases of non-infectious degeneration of a bioprosthetic valve. The presence of a valve stent ensures the stability of TAVI deployment without the risk of embolization. However, in such cases, the risk of CAO increases. Indeed, TAVI in native aortic valve stenosis is associated with a low risk of coronary obstruction (<1%) (2–6), while in valve-in-valve (ViV)-TAVI, this risk increases three- to fourfold (3, 4).

CAO following a TAVI procedure represents a devastating complication, with in-hospital mortality reaching up to 50%. CAO occurs three times more frequently in patients with degenerated surgical bioprosthetic aortic valves. In most cases, CAO occurs (5) either by direct obstruction of the coronary ostia or by caging the sinus of Valsalva at the sino-tubular junction, resulting in sequestration of the sinus and blocking blood flow to the coronary arteries (5, 7).

The choice of bioprosthetic valves significantly impacts the risk of CAO. Stented bioprosthetic valves with externally mounted leaflets and non-stented valves account for up to 80% of obstructions. Factors such as a small bioprosthetic valve and baseline stenosis are associated with high mortality rates. High-risk obstruction anatomies can be estimated using the VIVID classification (8), which is based on residual free distances after virtual valve implantation on CT scans using 3Mensio software. This classification was recently validated by Tomii et al. in a cohort of 137 Swiss patients (9).

When surgical redo is not feasible, the BASILICA technique can prevent CAO during ViV-TAVI. It creates a tear in the leaflet in front of the coronary artery, improving blood flow (8, 10). However, BASILICA is complex and carries increased risk in severe heart failure. The double chimney technique is a simpler alternative, keeping both coronary ostia open with stents. Now, the chimney technique can nonetheless present issues in case of the need for a new coronary access. Considering the patient’s coronary condition and age, we deemed this risk to be acceptable.

## Conclusion

In conclusion, our case adds to the growing body of evidence supporting the efficacy of the chimney technique in managing high risk of coronary artery obstruction during valve-in-valve procedures.

## Data Availability

The raw data supporting the conclusions of this article will be made available by the authors, without undue reservation.
